# Molecular docking analysis of nuclear factor-κB and genistein interaction in the context of breast cancer

**DOI:** 10.6026/97320630015011

**Published:** 2019-01-31

**Authors:** Vidya Mukund, Santosh Kumar Behera, Afroz Alam, Ganji Purnachandra Nagaraju

**Affiliations:** 1 Department of Bioscience and Biotechnology, Banasthali University, Banasthali, RJ, 304 022, India; 2 Biomedical Informatics Centre,Regional Medical Research Centre (ICMR),Bhubaneswar 751023,Odisha,India; 3 Department of Hematology and Medical Oncology,Winship Cancer Institute, Emory University, Atlanta, GA, 30322, USA

**Keywords:** Breast cancer, NF-κB, Genistein, Docking

## Abstract

Nuclear factor kappa-light-chain-enhancer of activated B cells (NF-κB) is a transcription factor and it contributes to breast cancer growth and metastasis. Hence, NF-κB is considered as a target for anti-breast cancer drugs. NF-κB was retrieved from the UniProtKB Data Base with UniProt ID P19838, its energy was minimized and subjected to molecular dynamic simulations using Gromacs v5.0.7 software with GROMOS96 43A1 force field implementing the steepest descent algorithm. The structure of genistein was retrieved from NCBI PubChem database in .sdf format and convert to .pdb format. The genistein compound was docked into the active site of NF-κB proteins with AutoDock tools 1.5. The genistein compound displayed the best binding energies at -6.29 (NF-κB) kcal/mol correspondingly. The binding interactions of this compound with the active site of NF-κB proteins suggested that amino acid residues (Lys52, Ser243, Asp274, Lys, 275) might play a key role in anti-breast cancer activity. Genistein also inhibited the translocation and expression of NF-κB in the nucleus of both breast cancer cell lines. These findings might increase our understanding of the molecular and functional role of NF-κB in breast cancer. It could also help in developing additional druggable NF-κB inhibitors with high potency, specificity and outstanding bioavailability.

## Background

Breast cancer is considered to be one of the most lethal disease in women and is the second leading cause of cancer-related deaths in the United States [1]. Nuclear factor kappa-light-chain-enhancer of activated B cells (NF-κB) is an important gene regulator, which plays a vital role in activation and progression of many cancers including breast cancer [2]. Consequently, inhibiting the activities of NF-κB in breast tumorigenesis would serve as a novel therapeutic strategy in breast cancer treatment. Genistein is naturally occurring isoflavonoid, which is profoundly found in products originating from soybean, has been associated with a decreased incidence of breast tumor in women around the world [3]. Genistein is a chemopreventive agent, which competes with estradiol for binding with estrogen receptor as weak estrogen [4]. It also aids in regulating signal transduction as protein tyrosine kinase inhibitor and also inhibits cellular oxidative stress as well as angiogenesis [5,6]. Targeting NF-κB activity inhibition may serve as a novel approach through which, genistein suppresses the breast cancer growth and progression.

## Methodology

The sequence, structure and functional information of NF-κB1 were retrieved from UniProtKB Data Base with UniProt ID P19838 (NFKB1_HUMAN) and review of literatures. The length of the protein was 968 amino acids.

## Structure Retrieval:

The three-dimensional (3D) structure of NF-κB1 with PDB ID: 1SVC was downloaded from research collaboratory in structural bioinformatics, protein data bank (RCSB, PDB) (http://www.rcsb.org/pdb/home.do) with 2.60 Å resolution and 311 amino acids length (42-353). The co-crystallized DNA was removed through BIOVIA Discovery Studio 4.5 Visualizer. The structure of Genistein (CID: 5280961) as inhibitor was retrieved from NCBI PubChem database in .sdf format. Online simplified molecular input line entry system (SMILES) translator web server (https://cactus.nci.nih.gov/translate/) was used to convert this file into .pdb format as an input to AutoDock 4.2 (autodock.scripps.edu/) docking tool.

## Validation of the crystal structure:

**Figure 1:**
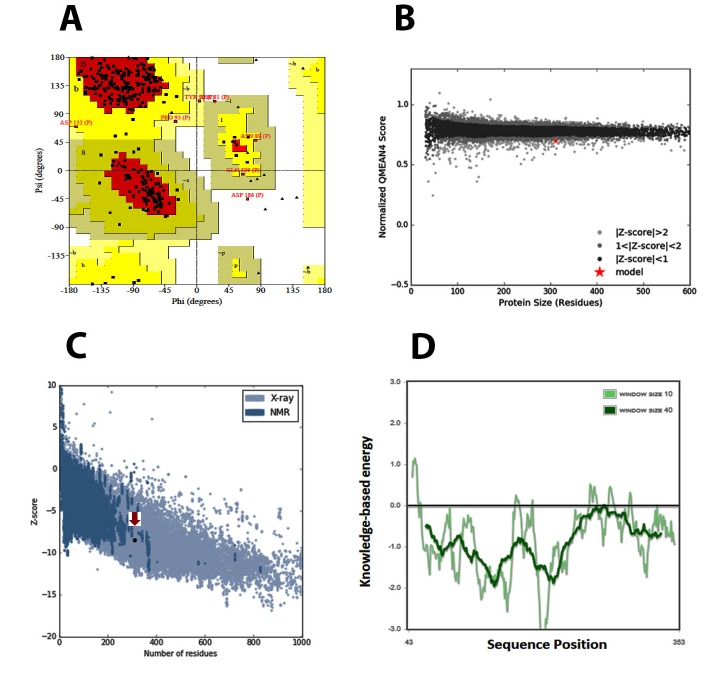
(A) Validation of the x-ray crystallography structure of NF-κB1 by Ramachandran plot. (B) Qmean based structure validation, which compares the structure to a non-redundant set of PBDs of similar size. The NF-κB1 structure, indicated using a red star, lies within the range of scores of similar size structures, indicating its good quality. (C) ProSa Z-score plot of the NF-κB1 is indicated with a red arrow. (D) The local quality of the model is shown in a plot of energy as a function of amino acid sequence position.

The quality of crystal structure coordinates was validated using several methods. The Ramachandran analysis [7] illustrated that 227 (85.3%) residues are in most favoured region, 33 (12.4%) are in additionally allowed region and 4 (1.5%) residues in generously allowed regions. There were 2 residues in outlier region (Figure 1A), which can be ignored. To check the quality of the crystal structure ProQ server was employed [8]. The server predicted LG score: 5.687 and MaxSub: 0.405 which depicted extremely good structure and fairly good structure. Using the qualitative model energy analysis (QMEAN) server [9], the model quality was determined. The overall quality of model was good as indicated by its QMEAN Z-score and QMEAN4 global score (Figure 1B). Low quality models are expected to have a negative QMEAN Z-score. The QMEAN4 ranges from 0 to 1 and a higher value indicates good quality model [9]. Additionally, the overall quality of the model was evaluated using Protein Structure Analysis (ProSA) tool (https://prosa.services.came.sbg.ac.at/prosa.php) which provides a quality score, Z-score as compared to all known protein structure from x-ray crystallography as well structural NMR [10]. The obtained Z-score value was -8.5, which indicates the high quality of the model compared to known protein structures (Figure 1C). The local quality of the model was also calculated and is presented in Figure 1D.

## Prediction of binding site:

A structural and active site study of NF-κB1 was carried out using computed atlas of surface topography of proteins (CASTp), GalxySite and bSiteFinder.

## Molecular Docking:

Molecular docking studies using NF-κB1 (energy minimized) with genistein as drug inhibitor was performed using AutoDock 4.2 tool. For protein, the Kollman charges were assigned and Geister partial charges were applied for inhibitor using AutoDock Tools 1.5. Docked structure with Genistein drug was compared with crystal structure to provide benchmarking. Docking parameters was selected by comparing the docked structure to the original crystallographic structure (PDB ID: 1SVC) and finding out similar binding modes. On the basis of grid dimension space was as follows: x-centering: 56.347, y-centering: 43.319 and z-centering: 49.667. The resultant docked poses were selected according to their binding energy scores, ligand efficiency and intermolecular H-bonds.

## Molecular Dynamics (MD) Simulations:

To understand the dynamic behaviour, mode of binding, and inhibitor specificity of NF-κB1, MD simulation was performed for one system viz., apo (only protein) using GROMOS96 43A1 force field in GROMACS 4.6.5 MD simulation package [11]. As the starting point, crystal structure of NF-κB1 protein was used as input. Finally, the energy-minimized system was subjected to position restrained simulation in constant number, volume and temperature (NVT) and isothermal-isobaric (NPT) ensemble. The NVT ensemble was used for temperature equilibration by restraining the positions of backbone atoms for 500 ps (pico second). NVT ensemble was followed by pressure equilibration using NPT ensemble for 500 ps. The final production MD runs were performed for 30ns with periodic boundary conditions keeping temperature of the systems 300 K. All the bond lengths were constrained using LINCS algorithm. Particle-mesh Ewald (PME) algorithm was used for long-range electrostatic interactions. 1.4 nm was set as the cutoffs for short-range electrostatic and van der Waals interaction.

The built-in modules of GROMACS and visual molecular dynamics (VMD 1.9.1) were employed to analyse the resultant trajectories. The 2D plots depicting the dynamics stabilities including root mean square deviation (RMSD), root mean square fluctuation (RMSF), and radius of gyration (Rg) of all systems were generated using Grace 5.1.23 program (http://plasma-gate.weizmann.ac.il/Grace/). Finally, Lig-Plot+, PyMOL, and Biovia Discovery Studio Visualizer v4.5 (BIOVIA, San Diego, CA) were used for image generation and receptor-ligand interaction analysis.

## Principal component analysis (PCA):

Principal component analysis (PCA) or Essential dynamics (ED) is a statistical method to separate the collective motions from the local dynamics by reducing high dimensional motional data sets into a small subset composed of principal components (PCs) that defines the collective motion. In order to identify the collective motion of the protein in apo state, we performed PCA by using gmx covera and gmx aneig tool in Gromacs. A set of eigenvectors and eigenvalues, which reflect concerted motion of the molecules, were extracted by diagonalizing and calculating the covariance matrix. The gmx anaeig tool was employed to analyze and plot the eigenvectors generated from the MD trajectory. In this study, the first two PCs, that is, PC1 and PC2 that dominates the collective motions in apo form of NF-?B1 were considered for analysis.

## Cell lines and culture:

MDA-MB-231 and T47D cell lines were obtained from ATCC (Manassas, VA) and are usually cultured at our facility. Genistein, and antibiotic solution, were obtained from Sigma Chemical Co. (USA). Antibodies specific for NF-κB, α-tubulin and lamin B were purchased from Santa Cruz Biotech (USA).

Cells were maintained in a humidified atmosphere (95% air+5% CO2) at 37°C. Cell lines were grown in Leibovitz's L-15 Medium or RPMI-1640 medium with 10% fetal bovine serum, 50 units/ml penicillin, and 50µg/ml streptomycin. 24h after seeding, cells were treated with 0.1% DMSO or 50µM of genistein. Cell lines were processed after 24hr of post-treatment for Western blot analysis.

## Sub-cellular fractionation:

Cytoplasmic and nuclear fractions were prepared using a nuclear extraction kit according to the manufacturer's instructions (Chemicon International, Inc., Temecula, CA). Briefly, harvested cells were lysed in cytoplasmic lysis buffer for 30min at 4°C and centrifuged (8000 x g for 20min). The supernatant was collected as the cytosolic fraction. The nuclear pellet was re-suspended in nuclear extraction buffer for 60min at 4°C and the nuclear fraction was collected after centrifugation (16,000 x g for 5min). Western blot analysis was performed for NF-κB, α-tubulin (cytoplasmic marker and loading control) and lamin B (nuclear marker and served as loading control).

## Results

### Molecular Docking

**Figure 2:**
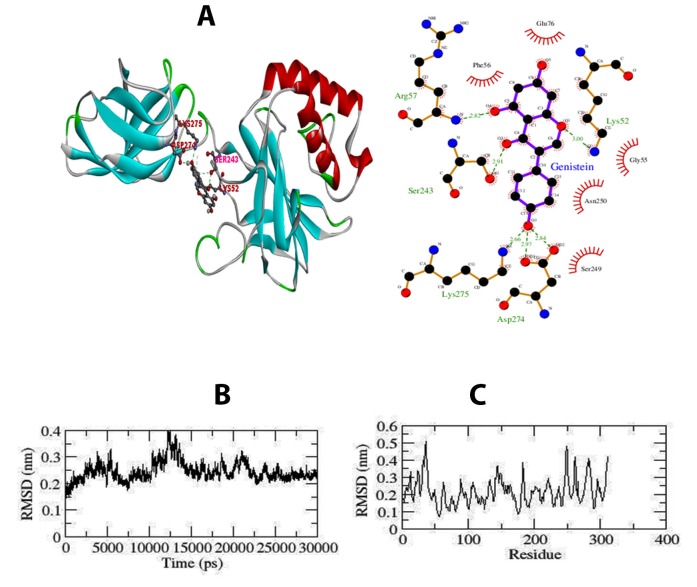
(A) 2D ligand interaction diagram created by LigPlot+ showing the hydrogen bond network and the hydrophobic interactions of genistein with the NF-κB1. (B) Conformational stability of apo form of NF-κB1 throughout 30 ns time period. Backbone-RMSD of NF-κB1 (Black color: Apo form) (C) Cα-RMSF profile of apo form of NF-κB1 during 30 ns MD (Black color: Apo form).

The docking methodology involves the extrapolation of ligand/inhibitor conformation and orientation within a targeted binding site or active site. The most popular docking tool AutoDock facilitated in predicting the activity of genistein against the NF-κB1. From the docking simulations, the promising pose with higher binding energy, ligand efficiency and intermolecular H-bonds was retained for detailed intermolecular interaction analysis. The predicted binding free energy of NF-κB1-genistein complex was found to be -6.29 kcal/mol (Table 1). In this study, we also examined the existence of intermolecular interactions in proteinligand complexes using LigPlot+ [12] programme and BIOVIA DS Visualizer (as shown in Figure 2A).

### Analysis of molecular dynamics simulation trajectory:

The Apo state of NF-κB1 was simulated using GROMACS version 4.6.5 for 30 ns. To gauge the dynamics and stability of each system, RMSD profile, Rg, Cα-RMSF, and intermolecular H-bonds were analyzed from the resultant MD trajectories using GROMACS utility toolkits. Having achieved a reasonable equilibrium, the results of these simulations are compared using a wide range of parameters and measurements and are detailed as follows:

### Analysis of molecular dynamics simulation trajectory:

Stability and Residue fluctuations: To access the dynamic stability of NF-κB1, RMSD profile for backbone residues were generated and plotted against time scale of 30 ns. As observed from the graph, the backbone RMSD of Apo state followed a deviation from 0.12 to ~0.23Å at 30 ns of simulation (Figure 2B). The Apo system possesses a significant structural deviation of Root Mean Square Deviation (RMSD) from starting to 25 ns, followed by a stable graph till 30 ns. We can conclude that binding of Genistein drug can stabilize structure of the NF-κB1 protein. To validate the results obtained from RMSD graph, we further calculated the fluctuation of residues using Root mean square fluctuation (RMSF). The RMSF of Ca-atom investigates the flexibility among the residues of NF-κB1protein.

In order to observe mobility of different residues, an RMSF plot was generated for Apo form (Figure 2C). Higher fluctuation pattern was observed in the Apo form demonstrating the restricted movements during the simulation. About 15 residues (250-265) displayed greater deviation in their Cα atoms in Apo system. With the support of MD trajectory file, the Cα-RMSF of each binding site residue was inferred from the NF-κB1-genistein complex. We have neglected 10 residues from both the ends of the protein. These terminal end residues often show high mobility because they reside at the ends of protein molecule. This inherent higher flexibility of amino acid residues indicates that these residues are crucial for ligand binding.

### Protein compactness and folding:

**Figure 3:**
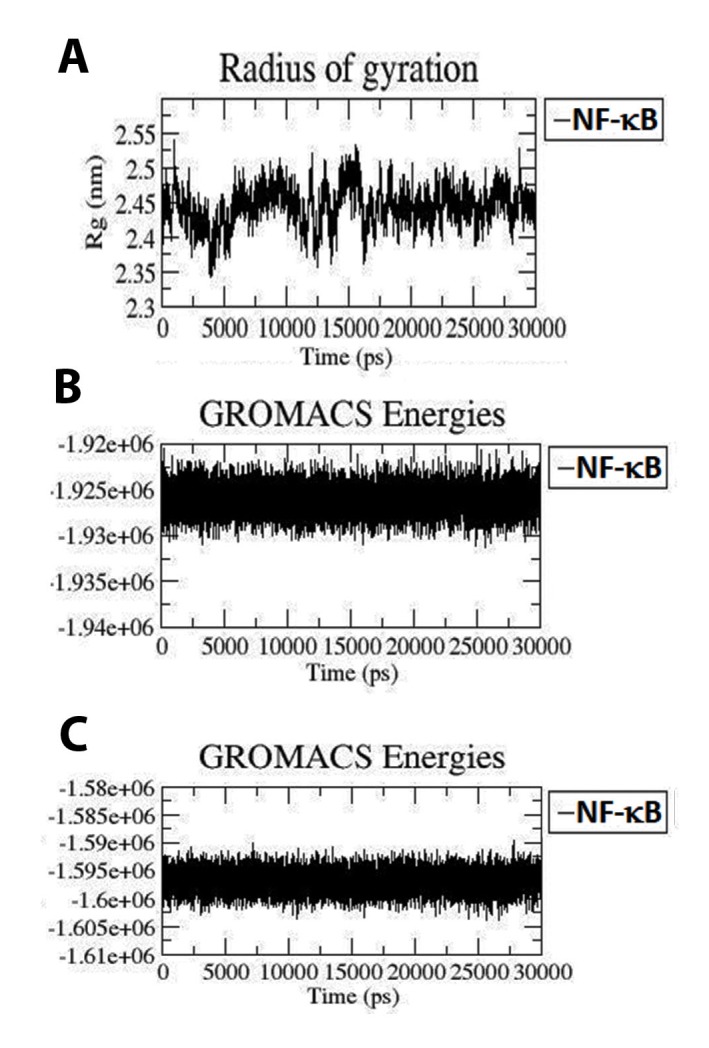
(A) Radius of gyration (Rg) profile of apo form of NF-κB1 during 30 ns MD (Black color: Apo form). (B) Potential energy of protein after 30ns simulation. (C) Total energy of protein after 30ns simulation.

To analyze the overall compactness of protein (apo form) an attempt was made to calculate the radius of gyration (Rg) values (i.e., mass weight root mean square distance for compilation of atoms from their common centre of mass) of both the systems. A relatively steady value of Rg over time shows that a protein is stably folded. To check this, radius of gyration vs. time graphs have been plotted for the structure (Figure 3A). After initial fluctuations, the Rg in both Apo state maintains a relatively steady value with an average value of 2.45 nm, this starts from 16 ns till the end of simulation time. Rg range of the Apo form is between 2.5 to 2.45 nm. This indicated that Apo is having better value of Rg. This indicates that Apo form increases its compactness through its advancement towards simulations. This result can be supported by analysis of the intermolecular hydrogen bond plot of complex form.

### Hydrogen bond analysis:

As the number of hydrogen bonds is related to binding strength, dotted lines were plotted to find out the number of hydrogen bonds between genistein and NF-κB1 (Figure 2A). H-bonds are indispensable for in molecular recognition as well as overall stability of a protein and its complex. Intermolecular hydrogen bonds were analysed for the protein-inhibitor complex system. The investigation illustrated that all the complexes displayed variable number of intermolecular hydrogen bonds (conventional hydrogen bond). The average distance of H bonds (Å) was 2.614 for NF-κB1- genistein complex. The change in intermolecular H-bonds and hydrophobic contacts observed in the complex perfectly correlate with the analysis of RMSF values indicating difference in residue flexibility of complex. Additionally, the 2D interactions of complex form showing the bonding pattern of genistein and its hydrophobic interactions created by LigPlot+.

### Energy analysis:

In addition to RMSD, stability of the protein and its complex was analysed through energy graphs. It is clear from Figure 3B and 3C that the potential and total energy of Apo form was found almost stationary during simulation which was confirmed by RMSD. Figure 3B depicted the potential energy of Apo form equivalent to - 1.925e+06KJ/mol (approx.), subsequently; Figure 3C depicted the total energy of Apo form equivalent to -1.595e+06KJ/mol (approx.). From the energy plots it can be concluded that the complex form can be found to be more stabilized then Apo as per the literature reviews.

### PCA:

**Figure 4:**
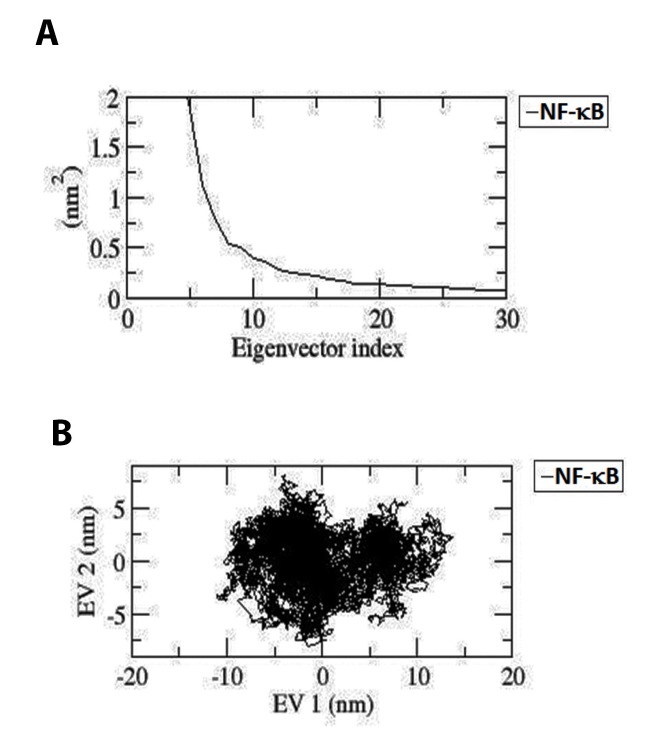
(A) Principal component analysis. The eigenvalues plotted against the corresponding eigenvector indices obtained from the Cα covariance matrix constructed from the 30 ns MD trajectory. First 30 eigenvectors were used for calculations. (B): Projection of the motion of the apo form of NF-κB1 in phase space along the first two principal eigenvectors (EV1 and EV2). The cloud represents the projection of trajectories of eigenvectors (EV1 and EV2) (Black color: apo).

The confined dynamical mechanical property i.e., fluctuation and structural motion of Apo state of NF-κB1 was investigated by using PCA or Essential dynamics (ED) analysis. The snapshots extracted at every two ps from the MD trajectories were projected onto space, which yielded a set of eigenvectors, which gives a vectorial representation of every solitary component of the motion revealing the direction of motion. As the first few eigenvectors capture bulk of the internal motions, the first two (EV1 and EV2) accounted for a noteworthy amount of overall motion in each case. The energetic participation of each component to the motion was obtained by analysing each eigenvector. Steep curves of eigenvalues (Figure 4A) were acquired after plotting eigenvalues against the eigenvectors, where, it was observed that 90% of the backbone motion was covered by the first 30 eigenvectors. The projection of trajectories obtained onto the first two principal components depicted the motion of Apo state of NF-κB1 protein in phase space (illustrated in Figure 4B). As Apo form depicted higher scattering of the atoms, which specify the occurrence of larger conformational changes in the crystal structure, which is in agreement with MD analysis.

The overall flexibility of Apo structure was calculated by the trace of the diagonalized covariance matrix of the backbone atoms. The trace values of covariance matrixes of Apo were found to be 47.8694 nm2, thus confirming the overall increased flexibility, which was well supported by RMSF analysis. The graph indicates the variance in the conformational distribution, where each dot represents one conformation of the protein.

### Genistein on NF-κB activation breast cancer cell lines:

**Figure 5:**
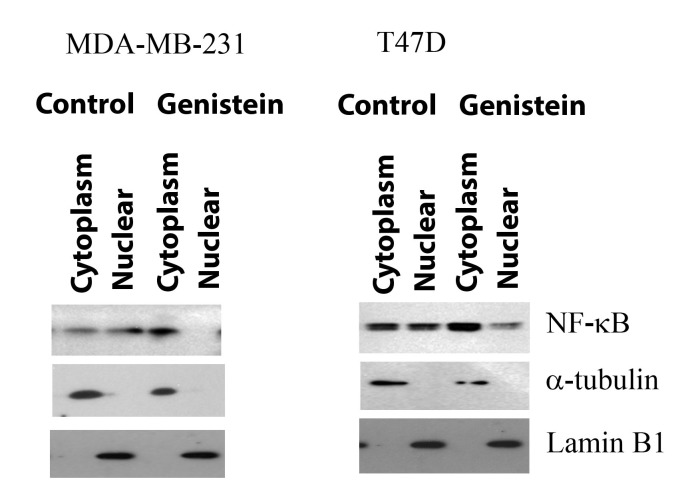
Genistein inhibits NF-κB translocation in breast cancer cell lines. MDA-MB231 and T47D cell lines were either untreated or treated with genistein (50µM) for 24h. Treatment with genistein decreased the nuclear translocation of NF-κB and increased the level of cytoplasmic NF-κB, as determined by Western blot.A- tubulin was used as the cytoplasmic marker, Lamin B1 as the nuclear marker.

Since NF-κB contributes to breast cancer growth and metastasis, we assessed the effect of genistein on this transcription factor.Treatment with ganetespib decreased NF-κB expression levels in the nucleus (Figure 5) and inhibited translocation of NF-κB to the nucleus, as shown in both breast cancer cell lines.

## Discussion

Genistein is known to inhibit tumor growth in numerous malignancies including breast cancer cell lines via G2/M cell cycle arrest and cyclin B1 downregulation [13]. It assists in inducing apoptosis by upregulating the expression of p21WAFI and Bax as well as stimulating CPP32 [2]. Genistein shows an inhibitory effect on invasion of breast cancer cells in vitro and downregulates MMP- 9 expression [14] and upregulates TIMP-1 expression [15]. In breast cancer cell lines, genistein can aid in activating NF-κB mediated apoptosis by generating reactive oxygen species (ROS) [16] and also inhibit activation of NF-κB via the MEK5/ERK5 pathway [17]. An investigation undertaken by a group revealed that human endometrial hyperplasial cell growth was significantly suppressed by genistein via EGFR inhibition as well as the inhibition of its downstream effectors NF-κB and PI3K/Akt [18].

As evident from the docking and MD simulation analysis, Genistein was understood as a better inhibitor of NF-κB1 that plays a key role in inhibition of breast cancer. The analysis has resulted in 4 conventional hydrogen bonds in NF-κB1-genistein complex, which depicted stronger interactions. The various plots, generated from MD simulations reflected the behaviour of amino acids residues of the Apo forms. The results from RMSD, RMSF and Rg to gather reflected the effect of NF-κB1 upon inhibition by genistein. The findings from the in silico analysis suggested genistein could serve as potential anti-cancerous drug for treatment of breast cancer. Further in vivo studies are necessary to confirm its efficacy and evaluate their anti-cancerous drug potency.

## Figures and Tables

**Table 1:** Molecular docking scores of Genistein participating in hydrogen bonding and other interactions with NF-κB1protein

Ligands/ Inhibitors	Binding energy (kcal/mol)	Ligand efficiency	Inhibition constant	No. of H bonds	Hydrophobic interaction forming residues	Average distance of H bonds (Å)
Genistein	-6.29	-0.31	24.52 µM	4 (Lys52, Ser243, Asp274, Lys275)	Lys52	2.614
